# Antigen-Bound and Free β-Amyloid Autoantibodies in Serum of Healthy Adults

**DOI:** 10.1371/journal.pone.0044516

**Published:** 2012-09-04

**Authors:** Madalina Maftei, Franka Thurm, Vera Maria Leirer, Christine A. F. von Arnim, Thomas Elbert, Michael Przybylski, Iris-Tatjana Kolassa, Marilena Manea

**Affiliations:** 1 Laboratory of Analytical Chemistry and Biopolymer Structure Analysis, Department of Chemistry, University of Konstanz, Konstanz, Germany; 2 Steinbeis Research Center for Biopolymer Analysis, University of Konstanz, Konstanz, Germany; 3 Department of Psychology, University of Konstanz, Konstanz, Germany; 4 Clinical and Biological Psychology, Institute of Psychology and Education, University of Ulm, Ulm, Germany; 5 Department of Neurology, University of Ulm, Ulm, Germany; 6 Zukunftskolleg, University of Konstanz, Konstanz, Germany; University of Quebect at Trois-Rivieres, Canada

## Abstract

Physiological β-amyloid autoantibodies (Aβ-autoantibodies) are currently investigated as potential diagnostic and therapeutic tools for Alzheimer’s disease (AD). In previous studies, their determination in serum and cerebrospinal fluid (CSF) using indirect ELISA has provided controversial results, which may be due to the presence of preformed Aβ antigen-antibody immune complexes. Based on the epitope specificity of the Aβ-autoantibodies, recently elucidated in our laboratory, we developed (a) a sandwich ELISA for the determination of circulating Aβ-IgG immune complexes and (b) an indirect ELISA for the determination of free Aβ-autoantibodies. This methodology was applied to the analysis of serum samples from healthy individuals within the age range of 18 to 89 years. Neuropsychological examination of the participants in this study indicated non-pathological, age-related cognitive decline, revealed especially by tests of visual memory and executive function, as well as speed-related tasks. The ELISA serum determinations showed significantly higher levels of Aβ-IgG immune complexes compared to free Aβ-autoantibodies, while no correlation with age or cognitive performance of the participants was found.

## Introduction

During healthy aging, a modest decline of fluid cognitive abilities (e.g., psychomotor speed, attention, short-term storage, verbal and visual episodic memory, visuospatial abilities and verbal fluency) can be observed, while crystallized cognitive functions such as semantic and procedural knowledge remain unimpaired [1]–[3]. Cognitive changes may start around the age of 20–30 years and progress until late adulthood with increasing interindividual variability [4], [5]. Healthy aging is further associated with biological changes, among which a decline in the specific immune response to antigenic stimuli was reported [6]. Age-related changes of the immune system are involved in the decreased response to vaccination, as well as in the susceptibility of elderly persons to infectious diseases and cancer [7], [8].

Physiological β-amyloid autoantibodies (Aβ-autoantibodies) have been identified in serum and cerebrospinal fluid (CSF) of healthy individuals and Alzheimer’s disease (AD) patients, as well as in human intravenous immunoglobulin preparations (IVIg), which are fractionated blood products used for the treatment of immune deficiencies and other disorders [9]. Du and colleagues found that Aβ-autoantibodies isolated from IVIg were able to block β-amyloid fibril formation and to inhibit β-amyloid-induced neurotoxicity in cultured rat hippocampal neurons [10]. Moreover, in a mouse model of AD, plaque formation was reduced after passive immunization with Aβ-autoantibodies and subsequent clearance of Aβ led to an improvement of mice behavior [11]. Considering that IVIg preparations contain Aβ-autoantibodies, they were used in small pilot trials for the treatment of AD patients [12], [13] and have been introduced into clinical trials as a potential AD treatment (www.clinicaltrials.gov; [12]). These results suggest a possible protective function of physiological Aβ-autoantibodies and raise the question whether low antibody levels might represent a risk factor for AD.

In order to evaluate the biomarker potential of Aβ-autoantibodies and to better understand their mechanism of action, several groups applied indirect ELISA protocols to determine the levels of Aβ-autoantibodies in serum or plasma of patients with AD or mild cognitive impairment (MCI). These previous studies have provided controversial results, since some groups reported lower levels of Aβ-autoantibodies in AD patients than in healthy individuals [14]–[17], while other groups found either increased levels [18] or no differences [19], [20]. Recently, Gustaw et al. [21] suggested that the detection of Aβ-autoantibodies in biological fluids was affected by the presence of Aβ peptides, and consequently of preformed Aβ-immune complexes. Using acidic dissociation of Aβ-IgG immune complexes and antigen removal prior to ELISA measurements, this group reported higher levels of Aβ-autoantibodies in serum of AD patients compared to healthy controls [21], [22]. However, using a similar procedure, Klaver et al. [23] found no significant differences between AD and control groups.

In the light of these conflicting results, an alternative approach would be the direct analysis of intact antigen-antibody immune complexes, which have been shown to be reliable biomarkers in various infectious diseases [24] and types of cancer [25], [26]. In the present study, we determined (a) by sandwich ELISA the levels of circulating Aβ-IgG immune complexes and (b) by indirect ELISA the free Aβ-autoantibody levels. The development of both ELISA methods was based on the evidence obtained in our laboratory indicating that “fibril-inhibiting” Aβ-autoantibodies recognize an Aβ(21–37) epitope [27], [28], in contrast to the “plaque-specific” antibodies produced by immunization, which bind Aβ(4–10) epitope [29], [30]. Thus, to capture the Aβ-IgG immune complexes from serum, we used a monoclonal antibody against the N-terminal Aβ-epitope for sandwich ELISA. To determine the levels of free Aβ-autoantibodies by indirect ELISA, biotinylated Aβ(12–40) epitope peptide was employed as capture antigen on streptavidin coated plates. Using these methods, serum samples from healthy individuals within the age range 18–89 years were analyzed. The main goals of this study were (1) to establish novel ELISA methods for the determination of intact Aβ-IgG immune complexes and free Aβ-autoantibodies and (2) to investigate whether serum levels of antigen-bound and free Aβ-autoantibodies correlate with age and cognitive status and thus could serve as a potential early indicator of an age-associated cognitive decline.

## Materials and Methods

### Materials

Mouse monoclonal 6E10 antibody (mAb 6E10) was purchased from Covance (Emeryville, California, USA), whereas human serum IgG preparations were obtained from Calbiochem (Merck, Darmstadt, Germany) and Talecris Biotherapeutics (Frankfurt am Main, Germany). Streptavidin, hydrogen peroxide and *o*-phenylenediamine dihydrochloride (OPD) were Merck products (Darmstadt, Germany), while horseradish peroxidase (HRP) labeled goat anti-human IgG (H+L) antibody was purchased from Pierce (Rockford, IL, USA). Bovine serum albumin (BSA) was a PAA Laboratories GmbH product (Pasching, Austria) and Tween-20 and Triton X-100 were obtained from Sigma-Aldrich (Steinheim, Germany). Costar 96-well ELISA plates were purchased from BioRad Laboratories (Munich, Germany).

### Ethics Statement

This study was approved by the ethics committee of the University of Konstanz and conducted according to the guidelines outlined in the Declaration of Helsinki. Prior to participation, written informed consent was obtained. All participants were cognitively healthy and able to consent (Mini Mental State Examination >25). Each subject received 30 Euro compensation for participation.

### Participants, Neuropsychological Examination and Serum Samples

Forty-seven healthy adults (21 males, 26 females) aged 18 to 89 years (*M* = 51.7, *SD* = 20.54) took part in this study. Educational level ranged from 10 to 21 years (*M* = 14.9, *SD* = 3.19) and was not associated with age (*r* = −0.16, *p* = 0.29). Sample details are depicted in Table 1.

**Table 1:** Means (*M*) and standard deviations (*SD*) of demographic data, MMSE scores and levels of Aβ-IgG immune complexes and free Aβ-autoantibodies (*n* = 47).

Sample characteristics	*M*	*SD*	*Range*
Age (years)	51.7	20.54	18–89
Education (years)	14.9	3.19	10–21
MMSE	29.5	0.95	26–30
Aβ-IgG levels (OD)^a^	0.596	0.24	0.09–0.99
Free Aβ-autoantibodies levels (OD)^a^	0.175	0.06	0.09–0.34

*Note.* OD* = *optical density (450 nm); MMSE – Mini Mental State Examination (CERAD-NP-plus; range 0–30);

a *n = *39.

Subjects were recruited in Konstanz, Germany, by notifications at the University of Konstanz, in public clubs, in senior citizen centers and in residential homes for the elderly as well as in local newspapers and radio stations. Exclusion criteria comprised: psychiatric disorders, a history of psychopharmacological medication, a history of severe head injuries or neurological problems (including epilepsy, stroke and brain tumors), dementia (according to DSM-IV-TR; [31]) or mild cognitive impairment in old age [32], [33]. During assessment, only 17 out of 47 participants (aged 18–89, *M* = 54.7, *SD* = 25.19) took at least one of the following types of medications: antihypertensive drugs (*n* = 5), thyroid hormones (*n* = 3), anti-inflammatory and analgesics (*n* = 5), antirheumatic medication (*n* = 1), cortisol (*n* = 1), cholesterol-lowering medication (*n* = 3), antihistamines (*n* = 2), prostate medication (*n* = 2) and hormones or contraceptives (*n* = 4). Thirteen of these participants took only one type of medication; four participants (2 males, 2 females) took three or four types of medication (aged 73, 75, 82 and 87 years).

Psychiatric disorders were assessed using the Mini International Neuropsychiatric Interview (M.I.N.I., German version 5.0.0 for DSM-IV; [34]). The subsequent neuropsychological examination included the following tests and test batteries: first, the Consortium to Establish a Registry for Alzheimer’s disease (CERAD-NP-plus) test battery [35] was used, namely subtests Mini Mental State Examination (MMSE), Boston naming test, semantic and phonemic fluency, word list learning, word list delayed recall, word recognition, figure copy, figure recall and trail making test (TMT) A and B. In addition, the German Wechsler Adult Intelligence Scale (HAWIE-R; [36]) was conducted, namely the subtests digit-symbol substitution test, mosaic test and the digit span test. Finally, the German version of the revised Benton visual retention test [37] was applied. The participants in this study showed non-pathological, age-related cognitive decline, especially in speed-related tasks and tasks of executive function (e.g., TMT, digit-symbol test), as well as in visual memory (e.g., Benton test; see Table S1 in the supporting information). As expected, in a cognitively healthy group, almost no variance in the scores of the following tests was observed: MMSE, Boston naming test, word recognition test and figure copy test.

Blood samples were taken between 8∶30 and 11∶00 o’clock in the morning. Serum was obtained by centrifugation of the blood samples for 4 min at 2700 g. In order to investigate whether the level of Aβ-IgG immune complexes changes with time, ten participants (five males, five females) aged 26 to 86 years (*M* = 52.1, *SD* = 18.48) donated blood three more times after the initial baseline assessment (time = 0, 1 and 4 weeks), each time between 8∶30 and 10∶00 o’clock in the morning. From each individual, blood samples were collected exactly at the same time and the same day of the week.

### Synthesis of Biotin-G_5_-Aβ(12–40) Epitope Peptide

Peptide Biotin-GGGGGVHHQKLVFFAEDVGSNKGAIIGLMVGGVV-NH_2_ (Biotin-G_5_-Aβ(12–40)) was synthesized in our laboratory on a NovaSyn TGR resin by 9-fluorenylmethoxycarbonyl/tert-butyl strategy, using a semiautomated Peptide Synthesizer EPS-221 (ABIMED, Langenfeld, Germany). The detailed synthetic protocol is presented in the supporting information (Protocol S1). The crude peptide was purified by RP-HPLC on a semipreparative C_4_ column. Purified peptide was characterized by analytical RP-HPLC and matrix assisted laser desorption ionization-Fourier transform ion cyclotron resonance mass spectrometry (MALDI-FTICR MS) as previously described [38]. The analytical RP-HPLC profile and MALDI-FTICR mass spectrum of the purified peptide are shown in the supporting information (Figure S1).

**Figure 1:**
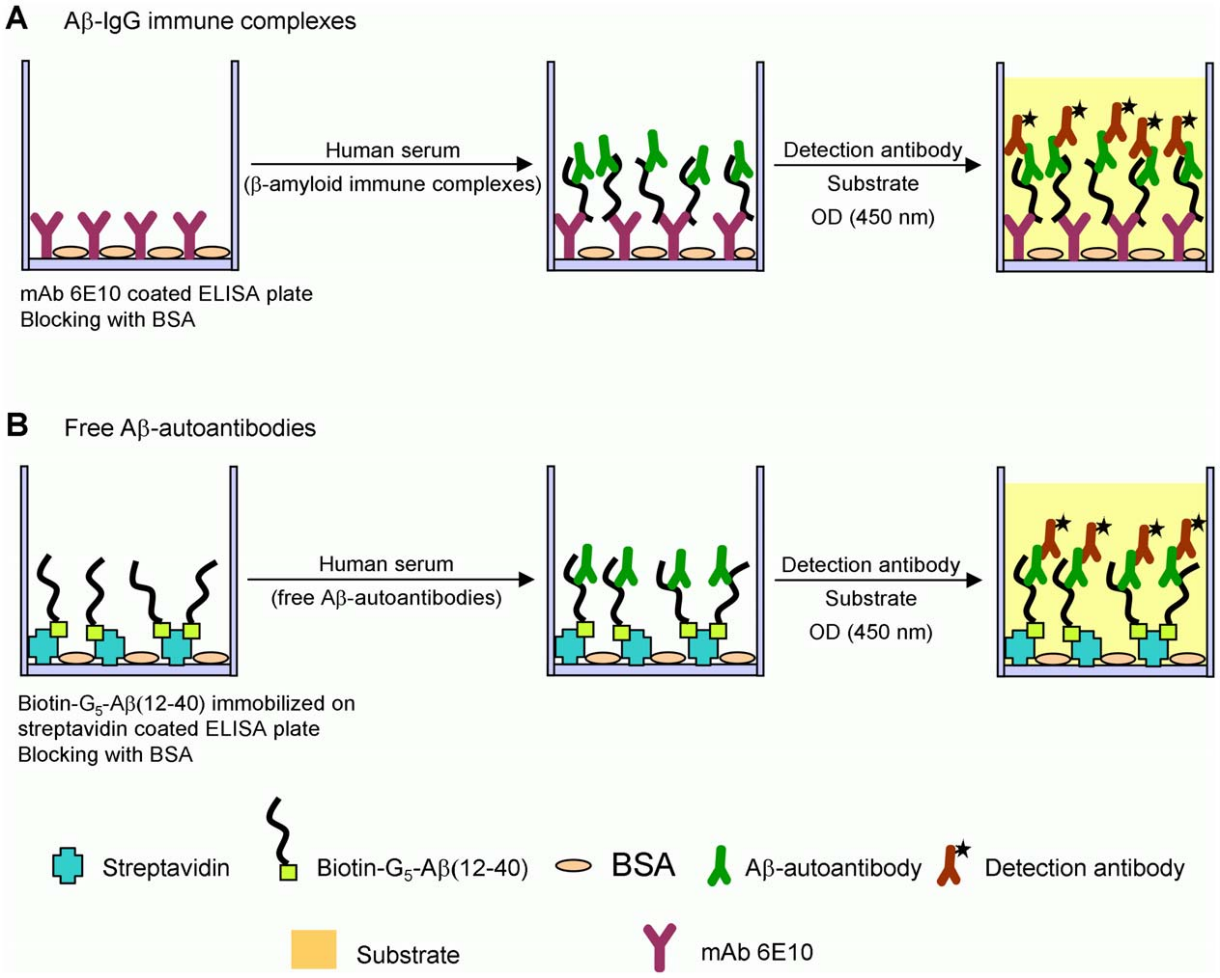
ELISA determination of β-amyloid immune complexes (Aβ-IgG) and free Aβ-autoantibodies in human serum. (A) Sandwich ELISA to determine Aβ-IgG immune complexes: mAb 6E10, recognizing Aβ(3–8) epitope, is first coated on the ELISA plates. After blocking with BSA, human serum containing β-amyloid immune complexes is added. The detection is performed with a labeled anti-IgG antibody. (B) Indirect ELISA to determine free Aβ-autoantibodies: Biotin-G_5_-Aβ(12–40) is immobilized on streptavidin coated ELISA plates. After blocking with BSA, the addition of serum containing free Aβ-autoantibodies leads to their binding to Aβ(12–40). The detection is performed with a labeled anti-IgG antibody.

### ELISA Determination of Aβ–IgG Immune Complexes in Serum

Costar 96-well ELISA plates were coated with 100 µL/well of mouse monoclonal antibody (mAb 6E10) at a concentration of 1 µg/mL [antibody solution prepared in phosphate buffered saline (PBS), pH 7.4] and incubated overnight at 4°C, followed by 30 min incubation at room temperature. The wells were washed four times with 200 µL/well washing buffer (0.05% Tween-20 v/v in PBS, pH 7.4), and then blocked with 5% BSA (w/v), 0.1% Tween-20 (v/v) in PBS. Following blocking, the plates were washed once with washing buffer and human serum samples were applied in triplicate (100 µL/well, 1∶100 dilution in blocking buffer) and incubated for 2 h at room temperature (RT). After washing the plates five times with washing buffer, 100 µL of horseradish peroxidase (HRP)-conjugated goat anti-human IgG (H+L) antibody diluted 5000 times in blocking buffer were added to each well. After incubation for 1 h at room temperature, followed by three times washing with washing buffer and once with citrate-phosphate buffer (0.1 M citric acid x H_2_O, 0.2 M Na_2_HPO_4_ x 2 H_2_O, pH 5.0), 100 µL/well of a mixture of *o*-phenylenediamine dihydrochloride in sodium phosphate-citrate buffer (c = 1 mg/mL) and hydrogen peroxide were added (2 µL of 30% hydrogen peroxide were used per 10 mL of substrate solution). The optical density (OD) at 450 nm was measured on a Wallac 1420 Victor^2^ ELISA Plate Counter (Perkin Elmer, Rodgau, Germany).

Human serum γ-globulin (immunoglobulin preparation, Calbiochem, Merck, Darmstadt, Germany) was used as reference in each experiment and it was applied in triplicate on each ELISA plate to allow data to be normalized between plates and different experiments. A stock solution of 7 µg/µL (approximating the average IgG level in serum of healthy individuals) in blocking buffer was prepared, diluted first 33.3 times and then three-fold serially (eight dilutions). Triplicate wells containing all components except the mAb 6E10 were used to assess the non-specific binding (NSB), both in the case of IgG preparation and serum samples. The average values, NBS subtraction, standard deviation (SD) and intra-assay and inter-assay coefficients of variation (CV) were calculated with the WorkOut2.0 software (Perkin Elmer, Rodgau, Germany).

**Figure 2:**
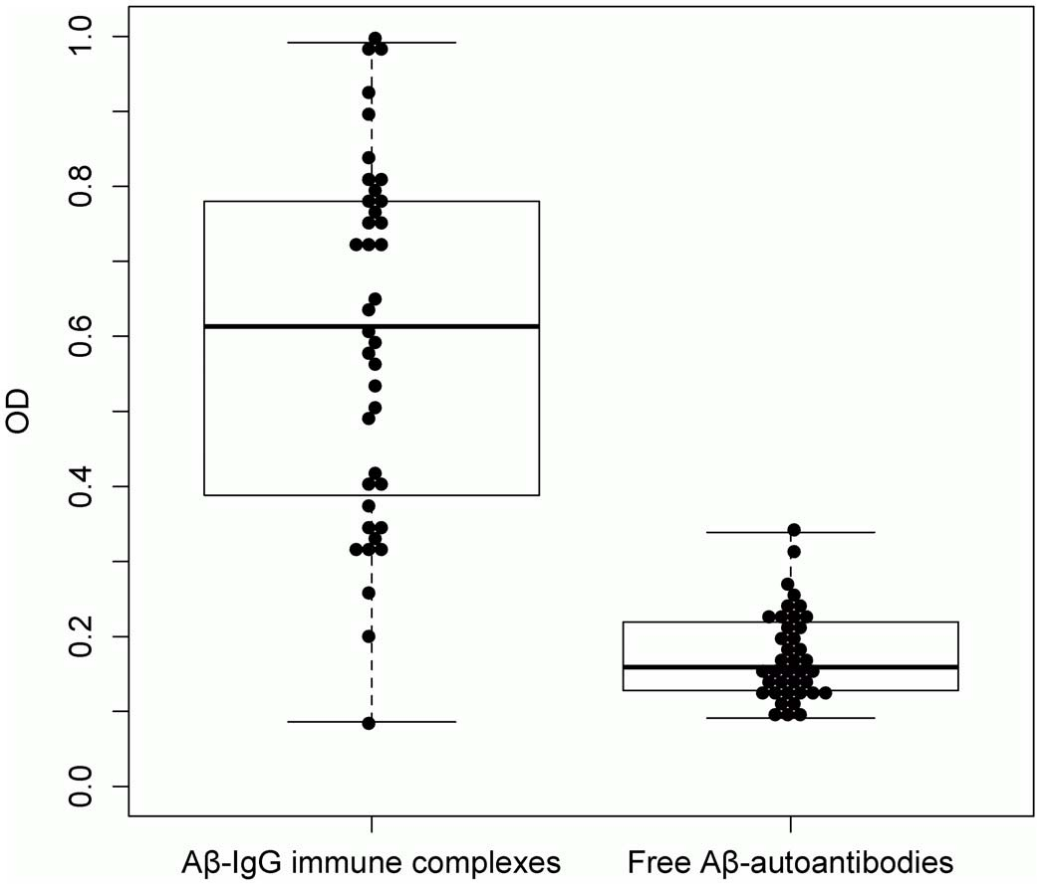
Levels of antigen-bound and free Aβ-autoantibodies (OD at 450 nm) in serum of healthy adults. The mean levels of Aβ-IgG immune complexes are significantly higher than those of free Aβ-autoantibodies; *t*_(35)_  = 10.12, *p*<0.0001.

### ELISA Determination of Free Aβ-autoantibodies in Serum

Costar 96-well ELISA plates were coated with 150 µL/well of streptavidin solution (c = 2.5 µg/mL in 5 mM Na_2_HPO_4_ and 150 mM NaCl, pH 7.4) and incubated overnight at 4°C, followed by 30 min incubation at room temperature. After washing the plates four times with 200 µL/well of washing buffer (0.05% Tween-20 in PBS, pH 7.4, v/v), 100 µL/well of Biotin-G_5_-Aβ(12–40) peptide (c = 2.5 µg/mL in PBS, pH 7.4) were added and incubated for 2 h at room temperature. After that, the wells were washed four times with 200 µL/well of washing buffer and blocked with 5% BSA (w/v), 0.1% Tween-20 (v/v) in PBS. Following blocking, the plates were washed once with washing buffer and human serum samples were applied in triplicate (100 µL/well, 1∶100 dilution in blocking buffer) and incubated for 2 h at RT. The next steps (washing, adding the detection antibody, optical density reading) were performed as described above. Human serum γ-globulin (Calbiochem, Merck, Darmstadt, Germany) was used as reference in each experiment and it was applied in triplicate on each ELISA plate as above mentioned.

**Figure 3:**
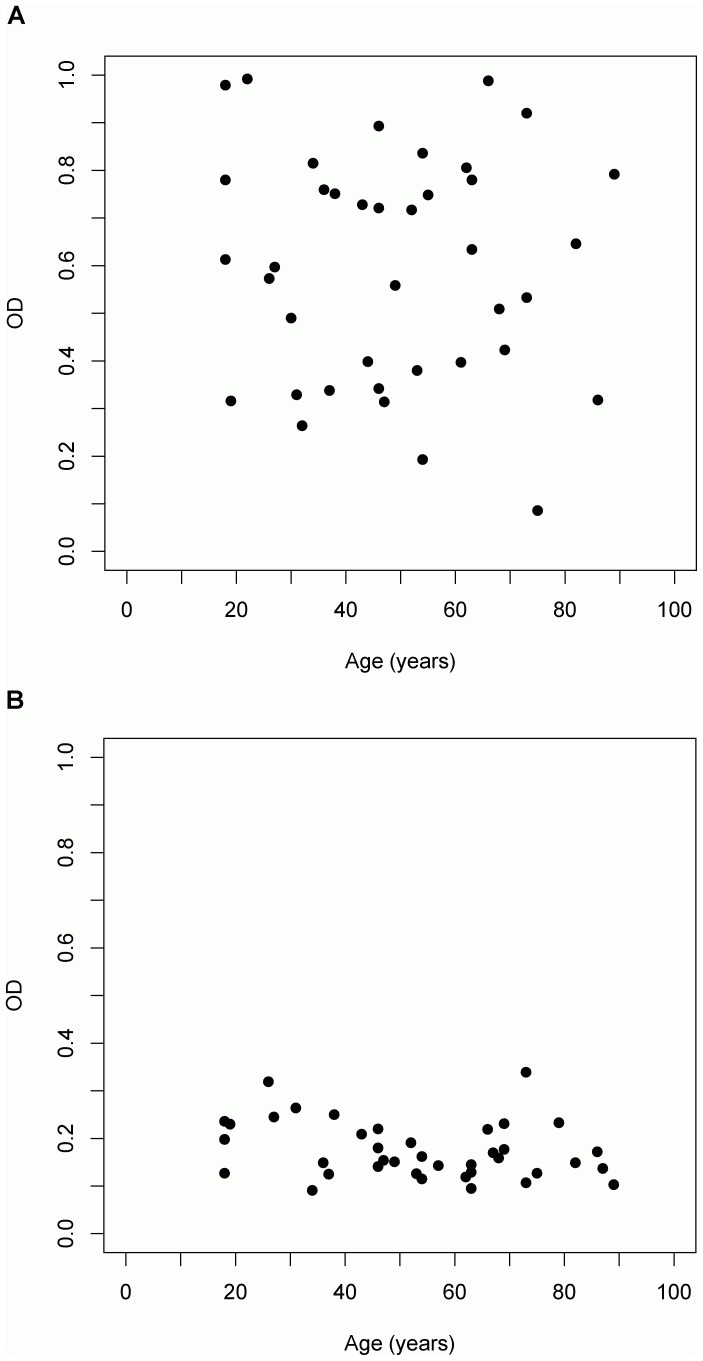
Correlation analysis between the age of healthy adults and serum levels of Aβ-autoantibodies. (A) Aβ-IgG immune complexes (OD at 450 nm; *r* = −0.08, *p* = 0.63) and (B) free Aβ-autoantibodies (OD at 450 nm; *r* = −0.28, *p* = 0.09).

### Statistical Analysis

Data were analyzed using R statistical software package of The R Foundation of Statistical Computing (www.r-project.org; version 2.11.1 for Mac OS X, GUI 1.34 Leopard).

Sample characteristics and the levels of Aβ-autoantibodies (level of free Aβ-autoantibodies vs. Aβ-IgG immune complexes) were calculated with Welch’s two-sample *t*-test (two-tailed with modified degrees of freedom). Possible correlations between Aβ-autoantibodies, age, neuropsychological test scores and years of education were computed with the Pearson's *r* product moment correlation coefficient. Since there was almost no variance (ceiling effect) in the test scores of MMSE, Boston naming test, word recognition test and figure copy test, these tests were not included into further analysis. *P*-values of multiple correlations were adjusted according to Holm’s sequential rejection algorithm [39]. The variation over time of Aβ-IgG immune complexes in serum was analyzed by mixed effects repeated measurement analysis of variance model (*F*-statistic) with a random intercept for participants (package *nlme* for R; [40]). Normality of the model’s residuals was tested using the Shapiro-Wilk normality test and visually inspected by residual density plot and Q-Q plot. All tests for statistical significance were applied with a significance level of *α* ≤0.05.

**Table 2:** Pearson’s *r* correlations between the levels of Aβ-IgG immune complexes (*n* = 39) versus free Aβ-autoantibodies (*n* = 39) and cognitive performance.

	Aβ-IgG immune complexes (OD)		Free Aβ-autoantibodies (OD)	
	*r*	*p*-value	*r*	*p*-value
Semantic fluency *	0.004	0.98	0.18	0.26
Phonemic fluency	0.23	0.17	−0.12	0.47
Word list learning **	−0.24	0.15	−0.15	0.37
Word recall **	−0.02	0.89	−0.16	0.32
Figure recall **	−0.003	0.99	0.09	0.57
TMT-A **	0.07	0.69	−0.18	0.27
TMT-B **	0.02	0.93	−0.17	0.30
Digit span test	0.04	0.79	0.20	0.23
Digit-symbol test **	−0.13	0.42	−0.004	0.98
Mosaic test **	−0.07	0.66	0.05	0.78
Benton test (correct) **	0.008	0.96	0.20	0.25
Benton test (error) **	−0.05	0.76	−0.17	0.32

Benton test (correct answers; range 0–20); Benton Test (errors; range 0–30); Digit span test (HAWIE-R; range 0–28); Digit-symbol substitution test (HAWIE-R; range 0–93); Figure recall (CERAD-NP-plus; range 0–14); Mosaic test (HAWIE-R; range 0–51); Phonetic/Semantic fluency (CERAD-NP-plus); TMT-A/B – Trail making test part A/B (CERAD-NP-plus; A: range 0–180 sec.; B: range 0–300 sec.); Word list learning (CERAD-NP-plus; range 0–30); Word recall (CERAD-NP-plus; range 0–10).

* Significant correlation between cognitive test performance and age.

** Significant correlation between cognitive test performance and age after correction for multiple correlation coefficients according to Holm.

**Figure 4:**
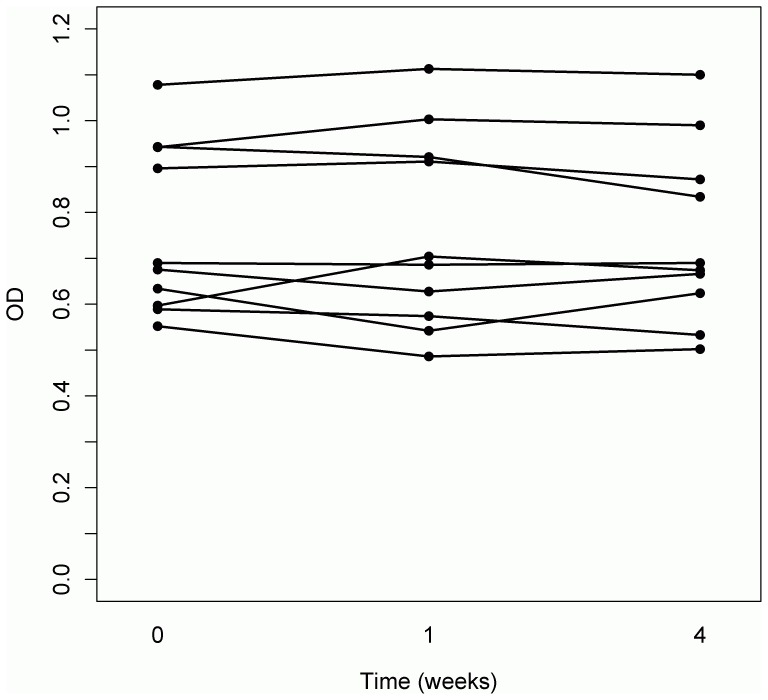
Serum levels of Aβ-IgG immune complexes (OD at 450 nm) over a four weeks period. Comparison between mean levels of Aβ-IgG immune complexes (OD at 450 nm) in serum provided by ten healthy individuals at three different time-points over a four weeks period (*F*_(2,18)_  = 0.23, *p* = 0.80). Each of the ten curves represents one individual subject.

## Results

### Aβ-IgG Immune Complexes in Serum

A novel sandwich ELISA for the determination of Aβ-IgG immune complexes in serum was developed based on the differential epitope specificities of Aβ-autoantibodies, which recognize Aβ(21–37) and of a mouse monoclonal 6E10 antibody (mAb 6E10), which binds to Aβ(3–8). The principle of sandwich ELISA for the determination of Aβ-IgG immune complexes is schematically shown in Figure 1A. Briefly, mAb 6E10, which was used for capturing Aβ-bound autoantibodies, was first coated on the ELISA plates. After blocking with BSA, human serum containing Aβ-IgG immune complexes was added. For detection, a horseradish peroxidase-labeled IgG, which recognizes human IgG and has no cross-reactivity with mouse IgG, was employed. It is worth mentioning that only soluble species were determined by ELISA and no precipitation was observed while performing the experiments.

To establish the concentrations of both capture and detection antibodies giving the highest OD response, a simultaneous two-dimensional serial dilution (chessboard titration) was applied (Figure S2, supporting information). The composition of washing buffer and the number of washing steps after sample addition was also varied to establish the assay conditions providing the highest OD after NSB subtraction (Figures S3, supporting information). Finally,different preincubation conditions for the IgG reference prior to its addition to the ELISA plates were tested (Figure S4, supporting information). Using the optimized ELISA protocol described in Materials and Methods, we investigated the presence of β-amyloid immune complexes in two IgG preparations: (1) human serum IgG from Calbiochem, commercialized for research purposes only and (2) intravenous immune globuline (IVIgG; Gamunex^®^ 10%) from Talecris Biotherapeutics (Frankfurt am Main, Germany), in use for treatment of different infectious, inflammatory and autoimmune disorders. β-amyloid immune complexes were detected in both preparations, slightly higher levels being observed in the product from Calbiochem (Figure S5, supporting information). The latter was applied as reference on each plate in subsequent ELISAs for the analysis of serum samples, to allow data normalization between different plates and experiments.

Since there is no unique method for expressing ELISA responses and arbitrary units are derived from absorbance readings, we considered it adequate to present the results of antigen-bound and free Aβ-autoantibodies determinations as OD values. The ELISA determinations of Aβ-IgG levels in both reference and serum samples showed intra-assay CVs <10% and inter-assay CVs <15%. A sigmoidal (5-parameters logistic) mathematical model was applied for reference curve fitting, providing good fits (R^2^>0.98; Figure S6A, supporting information). Since changes in OD units reflect equal changes in the analyte levels only in the linear region of the assay response, the reference curves were evaluated for linearity, which was observed between 0.068–1.109 OD units (R^2^>0.97 for linear regression). All serum samples gave absorbance readings within this interval and were included in the statistical analysis. The lower limit of detection (LLOD) of the assay, defined as 3 SD above the absorbance readings of the blank samples (blocking buffer without serum) was 0.064, slightly below the minimal OD cut-off value for the linearity constraint. The final sandwich ELISA protocol was employed for the analysis of serum samples from 39 healthy individuals aged 18 to 89 years (*M* = 48.8, *SD* = 19.87), proving OD values between 0.09–0.99 (*M* = 0.596, *SD* = 0.24; Figure 2). All serum samples were used at a dilution of 1∶100, which gave responses within the linear domain of the reference curve.

The levels of Aβ-IgG immune complexes in serum did not correlate with the age of the investigated healthy individuals (*r* = −0.08, *p* = 0.63; Figure 3A), their cognitive test scores (Table 2) or years of education (*r* = 0.01, *p* = 0.94). The variation with time of Aβ-IgG levels in serum of ten subjects (five males and five females), aged 26 to 86 years (*M* = 52.1, *SD* = 18.48) was also investigated and stable values for the Aβ-IgG immune complexes during a time interval of four weeks (week 0, week 1, week 4; *F*_(2,18)_  = 0.23, *p* = 0.80; Figure 4) were observed.

### Free Aβ-autoantibodies in Serum

Considering that Aβ-autoantibodies recognize Aβ(21–37) epitope, we developed an indirect ELISA for the determination of free Aβ-autoantibodies in serum. The principle of the assay is schematically shown in Figure 1B. To prevent conformational changes that may occur during direct adsorption on the ELISA plates, biotin-G_5_-Aβ(12–40) epitope peptide was employed as capture antigen on streptavidin coated plates. Washing and blocking steps, the addition of the detection antibody and the OD reading were performed as in the case of ELISA determination of Aβ-IgG immune complexes. As above mentioned, human serum IgG preparation (Calbiochem) was used as reference.

The OD readings of free Aβ-autoantibodies in both reference and serum samples showed similar CVs as those obtained for the determination of Aβ-IgG immune complexes. The linear range of the reference curve, fitted to a 5-parameters mathematical model (R^2^>0.98), was between 0.084–1.059 OD units, above the calculated LLOD of 0.072 OD units (Figure S6B, supporting information).

The indirect ELISA was applied to determine the levels of free Aβ-autoantibodies in 47 serum samples, using the same 1∶100 dilution as for the measurements of Aβ-IgG immune complexes. Eight samples provided OD values beneath the established linear interval of the assay response and were excluded from the statistical evaluation, leaving 39 sera (OD values between 0.09–0.34; *M* = 0.175, *SD* = 0.06; Figure 2) from healthy individuals aged 18 to 89 years (*M* = 53.4, *SD* = 20.51) to be further analyzed. In both young and older healthy subjects, we observed low but detectable levels of free Aβ-autoantibodies, significantly lower than those of Aβ-IgG immune complexes (*t*_(35)_  = 10.12, *p*<0.0001; Figure 2). No correlation of free Aβ-autoantibodies with age (*r* = −0.28, *p = *0.09; Figure 3B) was found. There was also no correlation with any cognitive test score (Table 2) or years of education (*r* = 0.22, *p* = 0.18).

Finally, the ratio of serum levels of Aβ-IgG immune complexes and free Aβ-autoantibodies was calculated and showed no correlation with age (*r* = 0.15, *p* = 0.42; Figure S7, supporting information), cognitive performance (Table S2, supporting information) or years of education (*r* = −0.01, *p* = 0.94).

## Discussion

β-amyloid autoantibodies are currently investigated as potential therapeutic and diagnostic tools for Alzheimer’s disease. However, their determination by indirect ELISA in serum or plasma of AD patients and healthy individuals has provided controversial results, which may be explained by the fact that Aβ-autoantibodies are circulating both in free and antigen-bound form. It has recently been suggested that serum levels of Aβ-autoantibodies after acidic dissociation of the immune complexes are of significant diagnostic value [21], [22].

The main goals of the present study were (1) to establish novel ELISA methods for the determination of antigen-bound and free Aβ-autoantibodies in serum and (2) to investigate whether their levels correlate with age and cognitive status of healthy adults and thus might serve as a potential early indicator of an age-associated cognitive decline. The analysis of intact immune complexes as an alternative to acidic dissociation may provide valuable additional information on possible problems related to antibody avidity and clearance of immune complexes. In both ELISA protocols, a commercially available IgG preparation was applied as reference, in order to normalize data between various plates and experiments. The sample-characteristic non-specific binding response was subtracted from the OD reading of each serum, a procedure previously reported only in a few ELISA studies of Aβ-autoantibodies, e.g. [23].

To our knowledge, this is the first report on the detection of intact Aβ-IgG immune complexes in human serum, as well as on the comparative determination of antigen-bound and free Aβ-autoantibodies in the same sample. Our data indicate that in serum of healthy adults aged 18–89 years, most of the Aβ-autoantibodies are bound to Aβ-peptides, forming Aβ-IgG immune complexes and only a small amount is circulating in free form (Figure 2). These results are in agreement with the publications reporting a significant increase of detectable levels of Aβ-autoantibodies upon acidic treatment of serum [21], [22]. The identification of circulating Aβ-IgG immune complexes in serum also provides a direct proof for the role of Aβ-autoantibodies in the binding and subsequent clearance of Aβ in vivo. The clearance mechanism of β-amyloid immune complexes is subject to further investigations.

The participants in the present study showed non-pathological, age-related cognitive decline, revealed especially in tests of visual memory and executive function, as well as in speed related-tasks (Table S1; see also [3]). Independent of age, participants were in a good health condition (only 17 participants took medication and only four of them more than one type of medication). We found no correlation between age or cognitive performances of healthy adults and the serum levels of Aβ-IgG immune complexes, free Aβ-autoantibodies or their ratio.

In conclusion, these data indicate that healthy aging per se is not associated either with an altered production of Aβ-autoantibodies or with an altered antigen-binding avidity, as reported in the case of AD patients [41]. The balanced formation and removal of the immune complexes in healthy individuals is also supported by the observed stability of Aβ-IgG immune complexes in serum over the investigated time period of four weeks. According to these results, serum levels of antigen-bound and free Aβ-autoantibodies are not associated with age or cognitive functions of healthy adults.

## Supporting Information

Figure S1 **Analytical RP-HPLC profile and MALDI-FT-ICR mass spectrum of Biotin-G_5_-Aβ(12–40) peptide.**
(PDF)

Figure S2 **Chessboard titration sandwich ELISA for determining the optimal concentrations of capture and detection antibodies.** The highest OD response (after NSB subtraction) was obtained using 1 µg/mL mAb 6E10 and 0.2 µg/mL HRP-conjugated goat anti-human IgG. The ELISA curves were drawn using the Excel software.
(PDF)

Figure S3 **Optimization of the washing buffer composition and number of washing steps in sandwich ELISA.** (A) Influence of the washing buffer composition and number of washing steps on the OD response (mean ± SD of three determinations) in sandwich ELISA, using 1/33.3 and 1/100 dilutions from an IgG stock solution (7 µg/mL) as test analyte. The coefficient of variation (CV) of the OD values obtained for each IgG dilution under the tested experimental conditions was below the accepted upper limit for the intra-assay CV (10%), indicating no significant differences. Highlighted with a red circle are the finally applied conditions for the determination of β-amyloid immune complexes; (B) Effect of the washing buffer composition and number of washing steps on the OD response (mean ± SD of three determinations) in sandwich ELISA, using 1/33.3 and 1/100 dilutions from a serum sample as test analyte. The coefficient of variation of the OD values obtained for each serum dilution under the tested experimental conditions was below the accepted upper limit for the intra-assay CV (10%), indicating no significant differences. Highlighted with a red circle are the finally applied conditions for the determination of β-amyloid immune complexes. PBST: 0.05% Tween-20 in PBS, pH 7.4 (v/v) PBS-Triton: 0.1% Triton X-100 in PBS, pH 7.4 (v/v) Composition of PBS: 137 mM NaCl, 2.7 mM KCl, 10 mM Na_2_HPO_4_ x 2 H_2_O, 2 mM KH_2_PO_4_ Composition of Cova buffer: 2 M NaCl, 1% MgSO_4_ x 7 H_2_O (w/w), 0.05% Tween-20 (v/v) in PBS.
(PDF)

Figure S4 **Influence of different preincubation conditions of the IgG reference on the ELISA response.** Different preincubation conditions of the IgG (Calbiochem) reference prior to its addition to the 6E10 antibody coated plates in sandwich ELISA led to almost identical results (CV_1/33.3_ = 7.49%, not significant). A 1 h incubation time at RT was chosen for further experiments. The ELISA curves were drawn using the Excel software.
(PDF)

Figure S5 **Comparison of the Aβ-IgG levels detected in two different IgG preparations:** IgG preparation (Calbiochem) and intravenous immune globuline (IVIgG; Gamunex^®^ 10%; Talecris Biotherapeutics). The ELISA curves were drawn using the Excel software.
(PDF)

Figure S6 **Examples of an IgG (Calbiochem) reference curve in (A) sandwich and (B) indirect ELISA.** In both cases, the IgG dilutions are plotted on a logarithmic scale and the corresponding OD readings (at 450 nm) fitted to a sigmoidal (5-parameters logistic) mathematical model using the WorkOut software. The triplicate OD readings for each IgG dilution are represented by red crosses. The linear range of each curve is highlighted in a blue box.
(PDF)

Figure S7 **Correlation analysis between the age of healthy individuals and the ratio of serum levels of Aβ-IgG immune complexes and free Aβ-autoantibodies (** ***r*** *=* **0.15,** ***p*** = **0.42).**
(PDF)

Table S1 **Means (** ***M*** **) and standard deviations (** ***SD*** **) of cognitive test scores.**
(DOC)

Table S2 **Pearson’s** ***r*** **correlations between the ratio of serum levels of Aβ-IgG immune complexes to free Aβ-autoantibodies and cognitive performance.**
(DOC)

Protocol S1 **Synthesis of Biotin-G_5_-Aβ(12–40) peptide.**
(PDF)
