# Correction: Antigen-Bound and Free β-Amyloid Autoantibodies in Serum of Healthy Adults

**DOI:** 10.1371/annotation/93ade307-e8d8-40f5-ad57-9e323548838b

**Published:** 2012-11-01

**Authors:** Madalina Maftei, Franka Thurm, Vera Maria Leirer, Christine A. F. von Arnim, Thomas Elbert, Michael Przybylski, Iris-Tatjana Kolassa, Marilena Manea

The Editor listed in the published manuscript is incorrect. The correct Editor is Dr. Heidar-Ali Tajmir-Riahi. His affiliation is the University of Quebec at Trois-Rivieres, Canada.

