# Supplementary material for: Antigen-Bound and Free β-Amyloid Autoantibodies in Serum of Healthy Adults
Source: PLoS One. 2012 Sep 4;7(9):e44516. doi: 10.1371/journal.pone.0044516 (PMC3433427; doi:10.1371/journal.pone.0044516)
Supplement: Figure S1 — Analytical RP-HPLC profile and MALDI-FT-ICR mass spectrum of Biotin-G5-Aβ(12–40) peptide. (PDF) [file pone.0044516.s001.pdf]

Biotin-G_5-VHHQKLVFFAEDVGSNKGAIIGLMVGGVV-NH_2

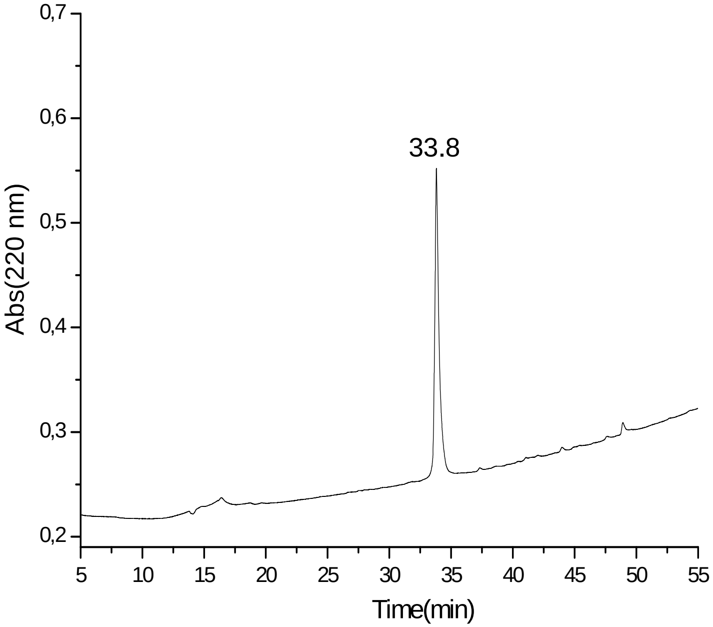

Vydac C_4 column
Eluents: 0.1% TFA in water (A), 0.1% TFA in MeCN: water 80:20 (B)
Gradient: 0 min 0% B, 5 min 0% B, 55 min 100% B

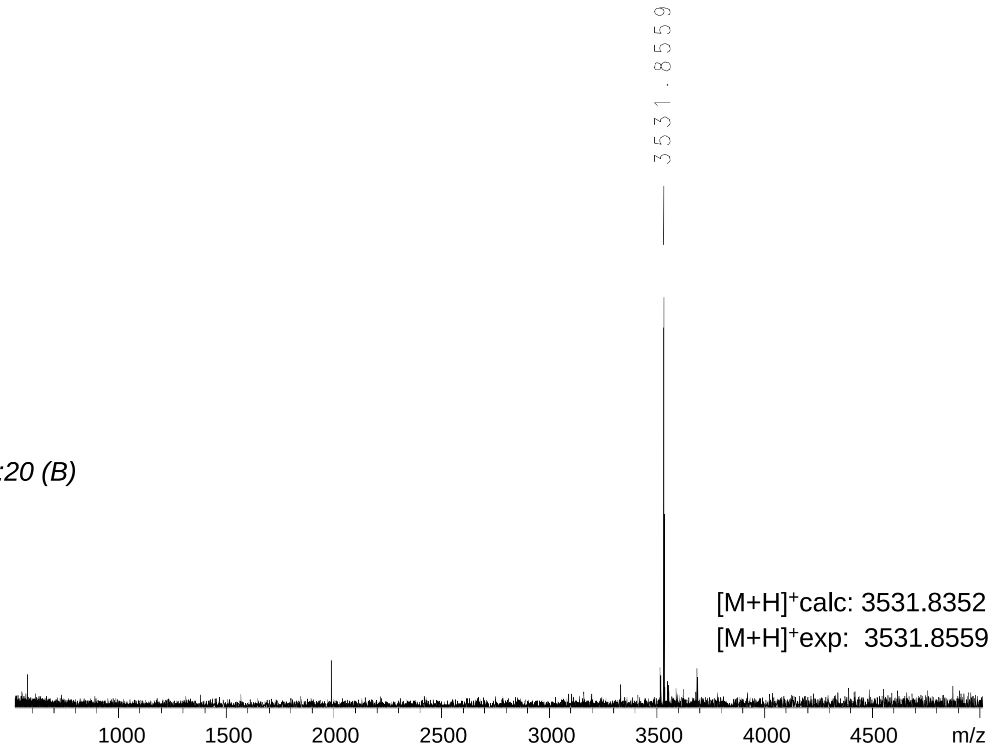

$[M+H]^+$calc: 3531.8352
$[M+H]^+$exp: 3531.8559
