# Supplementary figures and images for: Antigen-Bound and Free β-Amyloid Autoantibodies in Serum of Healthy Adults
Source: PLoS One. 2012 Sep 4;7(9):e44516. doi: 10.1371/journal.pone.0044516 (PMC3433427; doi:10.1371/journal.pone.0044516)

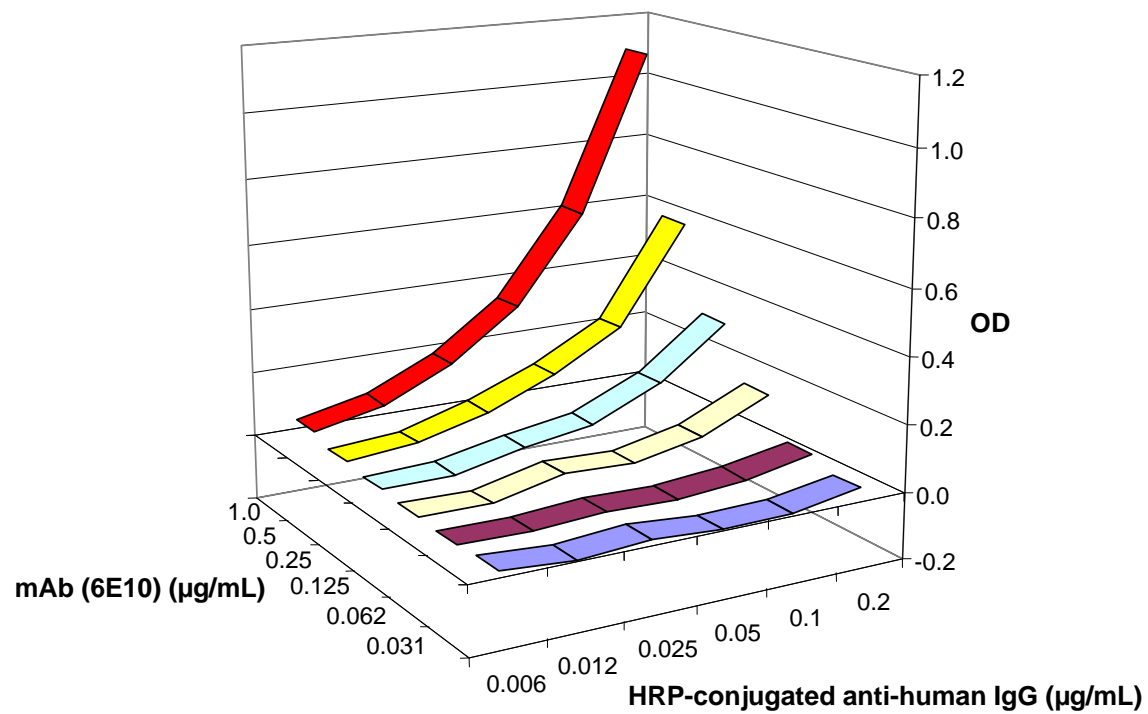

Supplement: Figure S2 — Chessboard titration sandwich ELISA for determining the optimal concentrations of capture and detection antibodies. The highest OD response (after NSB subtraction) was obtained using 1 µg/mL mAb 6E10 and 0.2 µg/mL HRP-conjugated goat anti-human IgG. The ELISA curves were drawn using the Excel software. (PDF) [file pone.0044516.s002.pdf]

**A**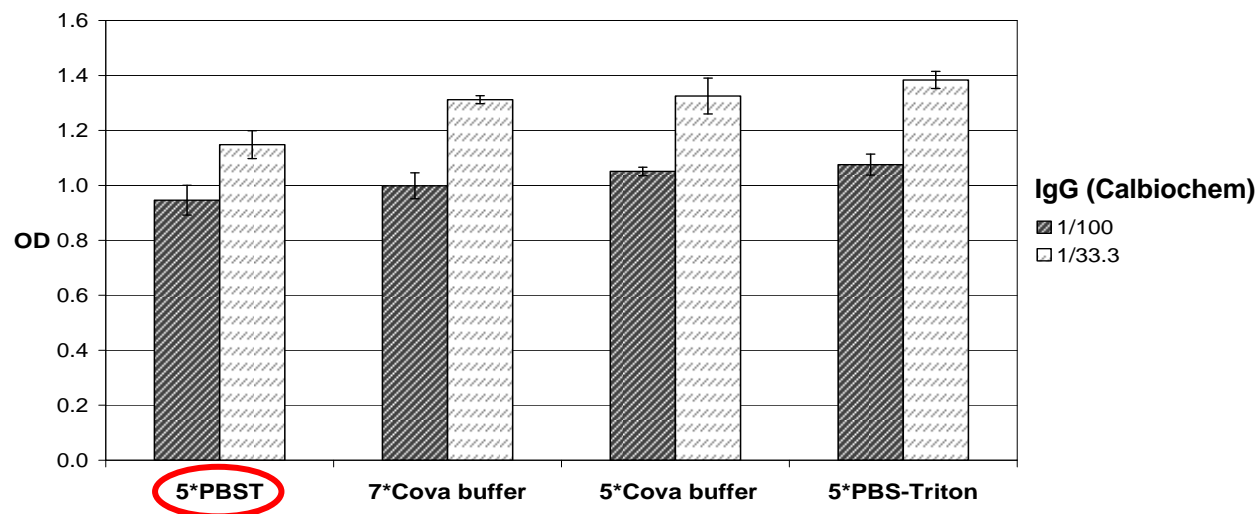**B**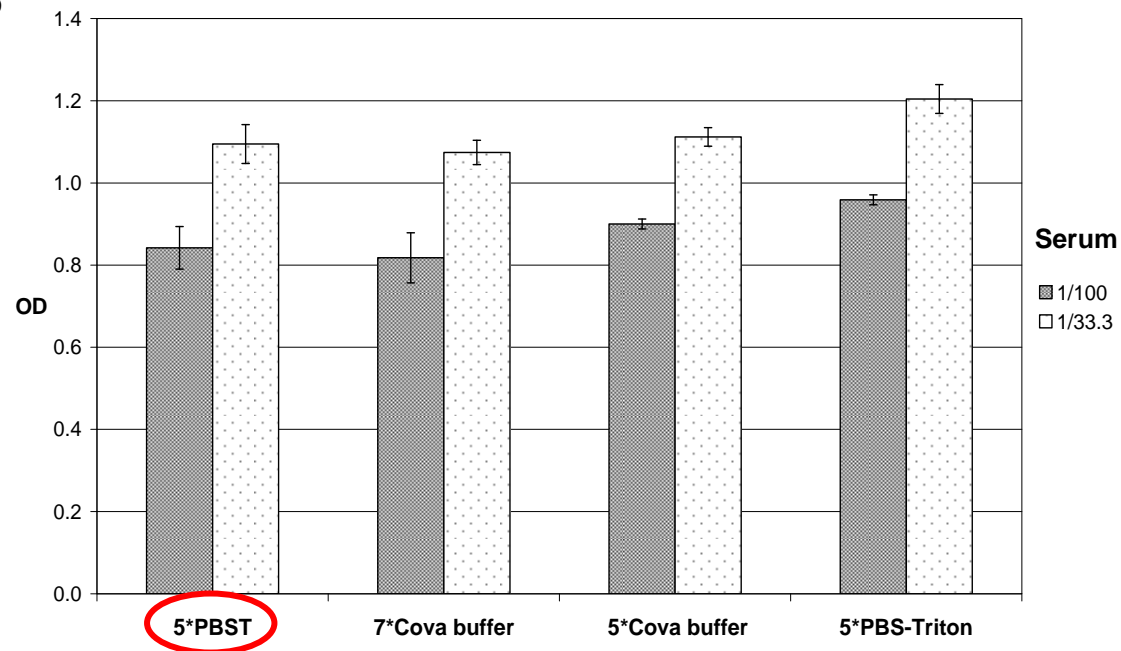

Supplement: Figure S3 — Optimization of the washing buffer composition and number of washing steps in sandwich ELISA. (A) Influence of the washing buffer composition and number of washing steps on the OD response (mean ± SD of three determinations) in sandwich ELISA, using 1/33.3 and 1/100 dilutions from an IgG stock solution (7 µg/mL) as test analyte. The coefficient of variation (CV) of the OD values obtained for each IgG dilution under the tested experimental conditions was below the accepted upper limit for the intra-assay CV (10%), indicating no significant differences. Highlighted with a red circle are the finally applied conditions for the determination of β-amyloid immune complexes; (B) Effect of the washing buffer composition and number of washing steps on the OD response (mean ± SD of three determinations) in sandwich ELISA, using 1/33.3 and 1/100 dilutions from a serum sample as test analyte. The coefficient of variation of the OD values obtained for each serum dilution under the tested experimental conditions was below the accepted upper limit for the intra-assay CV (10%), indicating no significant differences. Highlighted with a red circle are the finally applied conditions for the determination of β-amyloid immune complexes. PBST: 0.05% Tween-20 in PBS, pH 7.4 (v/v) PBS-Triton: 0.1% Triton X-100 in PBS, pH 7.4 (v/v) Composition of PBS: 137 mM NaCl, 2.7 mM KCl, 10 mM Na2HPO4 x 2 H2O, 2 mM KH2PO4 Composition of Cova buffer: 2 M NaCl, 1% MgSO4 x 7 H2O (w/w), 0.05% Tween-20 (v/v) in PBS. (PDF) [file pone.0044516.s003.pdf]

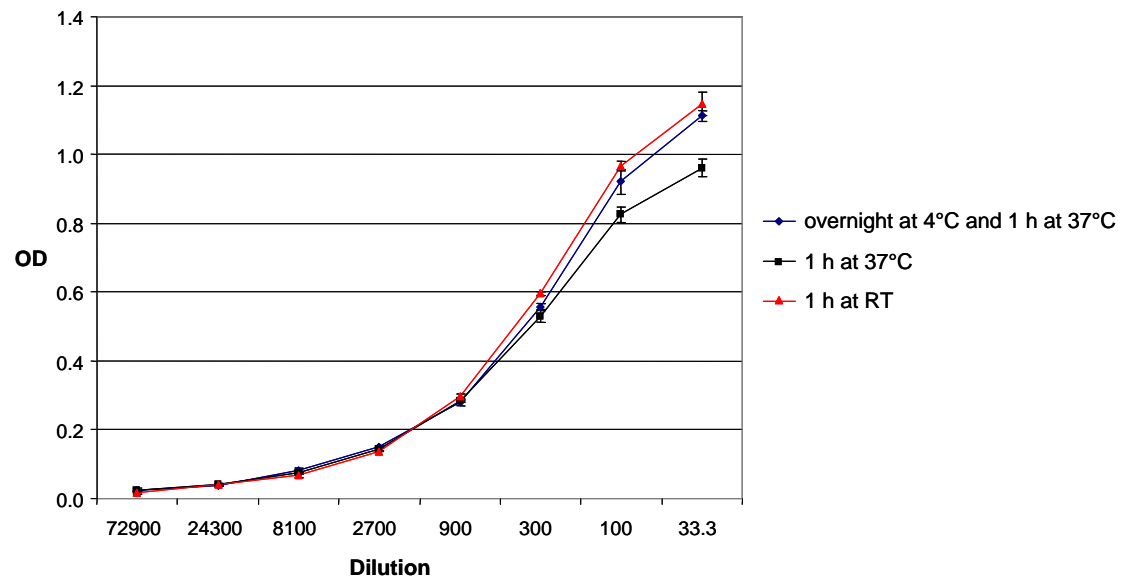

Supplement: Figure S4 — Influence of different preincubation conditions of the IgG reference on the ELISA response. Different preincubation conditions of the IgG (Calbiochem) reference prior to its addition to the 6E10 antibody coated plates in sandwich ELISA led to almost identical results (CV1/33.3 = 7.49%, not significant). A 1 h incubation time at RT was chosen for further experiments. The ELISA curves were drawn using the Excel software. (PDF) [file pone.0044516.s004.pdf]

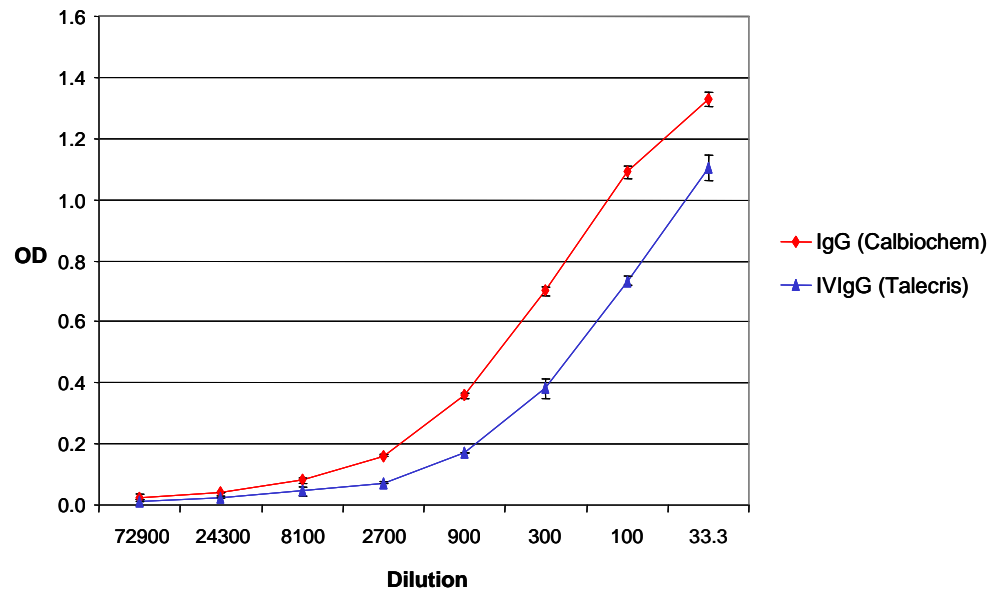

Supplement: Figure S5 — Comparison of the Aβ-IgG levels detected in two different IgG preparations: IgG preparation (Calbiochem) and intravenous immune globuline (IVIgG; Gamunex® 10%; Talecris Biotherapeutics). The ELISA curves were drawn using the Excel software. (PDF) [file pone.0044516.s005.pdf]

**A**Reference curve fit (5 parameters):  $R^2 = 0.9956$ 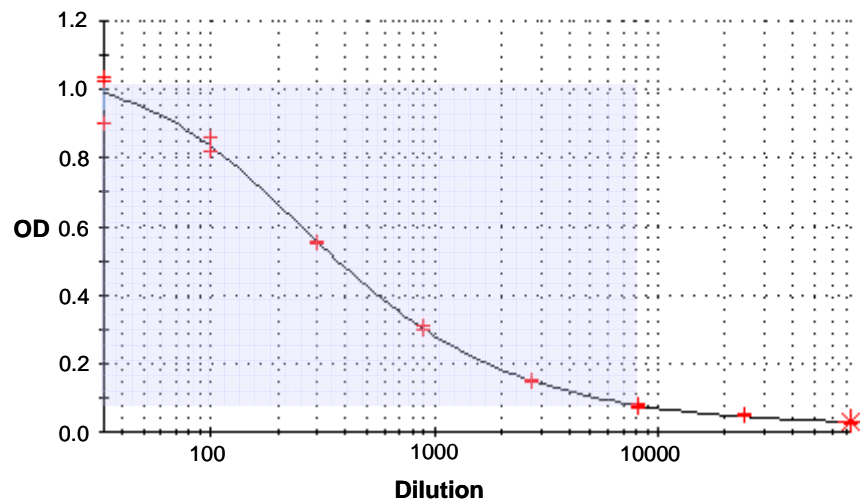**B**Reference curve fit (5 parameters):  $R^2 = 0.9972$ 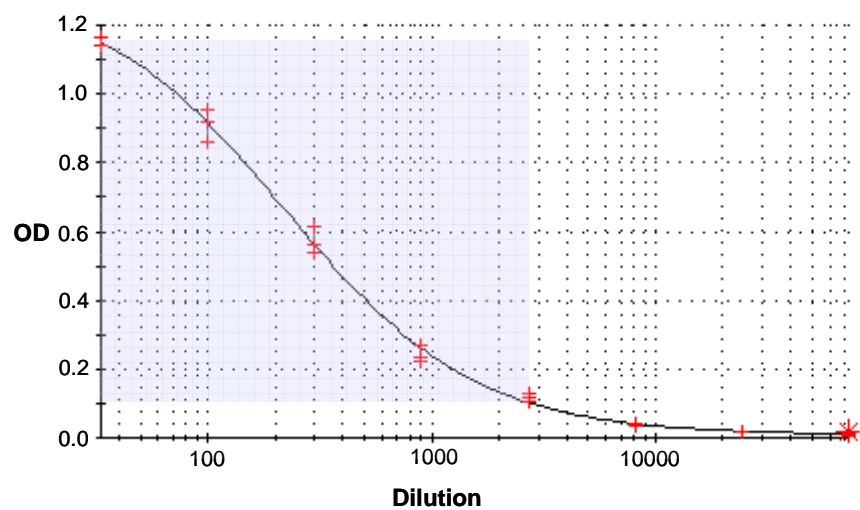

Supplement: Figure S6 — Examples of an IgG (Calbiochem) reference curve in (A) sandwich and (B) indirect ELISA. In both cases, the IgG dilutions are plotted on a logarithmic scale and the corresponding OD readings (at 450 nm) fitted to a sigmoidal (5-parameters logistic) mathematical model using the WorkOut software. The triplicate OD readings for each IgG dilution are represented by red crosses. The linear range of each curve is highlighted in a blue box. (PDF) [file pone.0044516.s006.pdf]

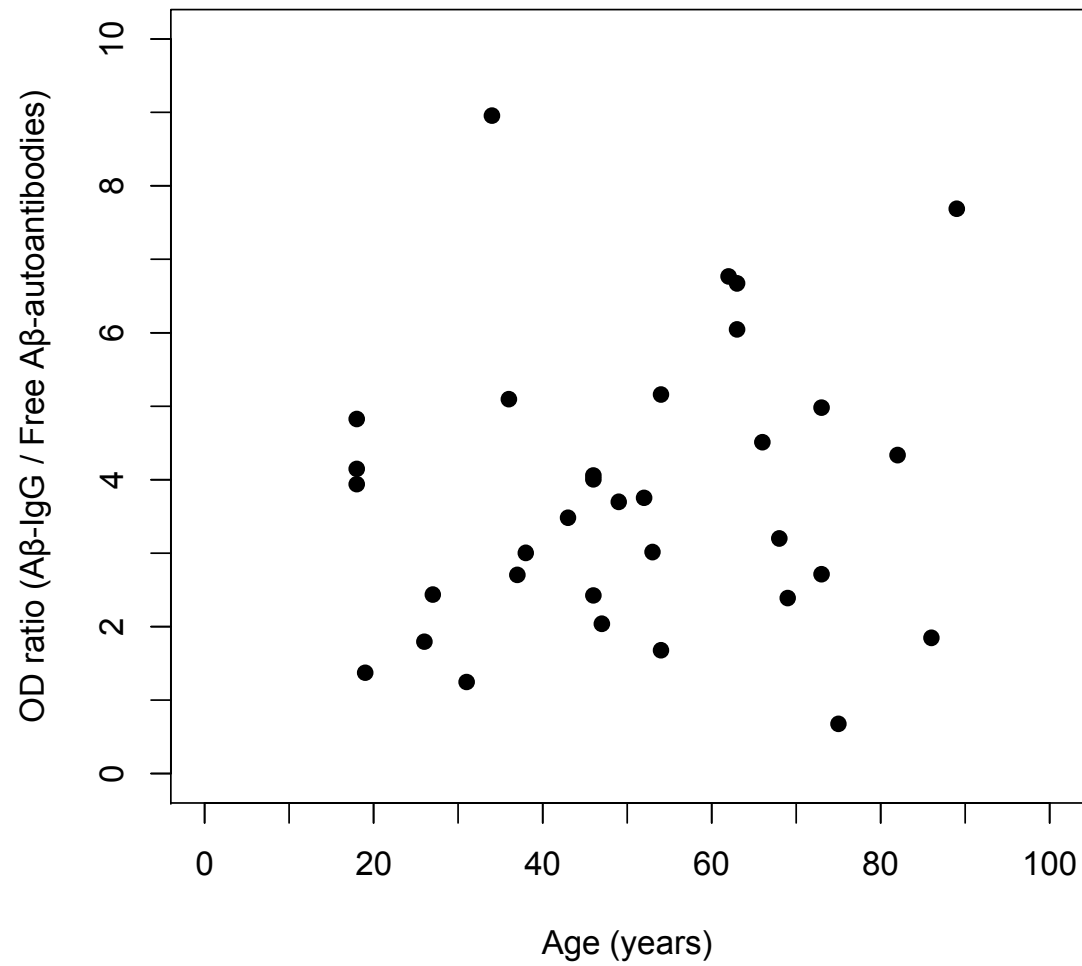

Supplement: Figure S7 — Correlation analysis between the age of healthy individuals and the ratio of serum levels of Aβ-IgG immune complexes and free Aβ-autoantibodies ( r = 0.15, p = 0.42). (PDF) [file pone.0044516.s007.pdf]
