# Supplementary material for: Antigen-Bound and Free β-Amyloid Autoantibodies in Serum of Healthy Adults
Source: PLoS One. 2012 Sep 4;7(9):e44516. doi: 10.1371/journal.pone.0044516 (PMC3433427; doi:10.1371/journal.pone.0044516)
Supplement: Table S1 — Means ( M ) and standard deviations ( SD ) of cognitive test scores. (DOC) [file pone.0044516.s008.doc]

| **Table S1.** Means (*M*) and standard deviations (*SD*) of cognitive test scores | | | | |
| --- | --- | --- | --- | --- |
| Cognitive test | | *M* | *SD* | *Range* |
| Boston Naming Test | | 14.7 | 0.66 | 12-15 |
| Semantic fluency * | | 24.1 | 5.55 | 13-38 |
| Phonetic fluency | | 15.3 | 5.23 | 4-27 |
| Word list learning ** | | 23.6 | 3.59 | 15-30 |
| Word recall ** | | 8.5 | 1.59 | 4-10 |
| Word recognition | | 9.9 | 0.37 | 8-10 |
| Figure copy | | 10.7 | 1.00 | 7-11 |
| Figure recall ** | | 11.8 | 2.32 | 6-14 |
| TMT-A ** | | 35.2 | 13.73 | 19-82 |
| TMT-B ** | | 81.2 | 44.70 | 35-270 |
| Digit Span Test | | 14.7 | 3.70 | 9-21 |
| Digit-Symbol Test ^a^ ** | | 53.4 | 13.10 | 28-80 |
| Mosaic Test ^a^ ** | | 33.4 | 10.03 | 8-50 |
| Benton Test (correct) ^b^ ** | | 13.2 | 4.09 | 4-20 |
| Benton Test (error) ^b^ ** | | 9.6 | 6.77 | 0-30 |
| *Note.* Benton Test (correct answers; range 0-20); Benton Test (errors; range 0-30); Boston Naming Test (CERAD-NP-plus; range 0-15); Digit Span Test (HAWIE-R; range 0-28); Digit-Symbol Substitution Test (HAWIE-R; range 0-93); Figure copy (CERAD-NP-plus; range 0-11); Figure recall (CERAD-NP-plus; range 0-14); Mosaic Test (HAWIE-R; range 0-51); Phonetic/Semantic fluency (CERAD-NP-plus); TMT-A/B – Trail Making Test part A/B (CERAD-NP-plus; A: range 0-180 sec.; B: range 0-300 sec.); Word list learning (CERAD-NP-plus; range 0-30); Word recall (CERAD-NP-plus; range 0-10); Word recognition (CERAD-NP-plus; range 0-10 true positives) | | | | |
| a  b  *  ** | *n* = 46  *n* = 44  Significant Pearson correlation between cognitive test performance and age  Significant Pearson correlation between cognitive test performance and age after correction for multiple correlation coefficients according to Holm | | | |
