# Supplementary material for: Antigen-Bound and Free β-Amyloid Autoantibodies in Serum of Healthy Adults
Source: PLoS One. 2012 Sep 4;7(9):e44516. doi: 10.1371/journal.pone.0044516 (PMC3433427; doi:10.1371/journal.pone.0044516)
Supplement: Table S2 — Pearson’s r correlations between the ratio of serum levels of Aβ-IgG immune complexes to free Aβ-autoantibodies and cognitive performance. (DOC) [file pone.0044516.s009.doc]

| **Table S2.** Pearson’s *r* correlations between the ratio of serum levels of Aβ-IgG immune complexes to free Aβ-autoantibodies and cognitive performance (*n* = 33) | | | |
| --- | --- | --- | --- |
|  | | Aβ-IgG / Free Aβ-autoantibodies ratio  (OD) | |
|  | | *r* | *p*-value |
| Semantic fluency * | | -0.19 | 0.28 |
| Phonemic fluency | | 0.32 | 0.07 |
| Word list learning ** | | -0.05 | 0.80 |
| Word recall ** | | 0.15 | 0.40 |
| Figure recall ** | | -0.09 | 0.63 |
| TMT-A ** | | 0.17 | 0.33 |
| TMT-B ** | | 0.17 | 0.35 |
| Digit span test | | -0.14 | 0.45 |
| Digit-symbol test ** | | -0.09 | 0.63 |
| Mosaic test ** | | -0.11 | 0.56 |
| Benton test (correct) ** | | -0.14 | 0.43 |
| Benton test (error) ** | | 0.05 | 0.78 |
| Benton test (correct answers; range 0-20); Benton test (errors; range 0-30); Digit span test (HAWIE-R; range 0-28); Digit-symbol substitution test (HAWIE-R; range 0-93); Figure recall (CERAD-NP-plus; range 0-14); Mosaic test (HAWIE-R; range 0-51); Phonetic/Semantic fluency (CERAD-NP-plus); TMT-A/B – Trail making test part A/B (CERAD-NP-plus; A: range 0-180 sec.; B: range 0-300 sec.); Word list learning (CERAD-NP-plus; range 0-30); Word recall (CERAD-NP-plus; range 0-10) | | | |
| ***  **** | Significant correlation between cognitive test performance and age  Significant correlation between cognitive test performance and age after correction for multiple correlation coefficients according to Holm | | |
