# Supplementary material for: Antigen-Bound and Free β-Amyloid Autoantibodies in Serum of Healthy Adults
Source: PLoS One. 2012 Sep 4;7(9):e44516. doi: 10.1371/journal.pone.0044516 (PMC3433427; doi:10.1371/journal.pone.0044516)
Supplement: Protocol S1 — Synthesis of Biotin-G5-Aβ(12–40) peptide. (PDF) [file pone.0044516.s010.pdf]

Protocol S1. Synthesis of Biotin-G_5-Aβ(12-40) peptide

All amino acid derivatives, benzotriazol-1-yl-oxytrispyrrolidinophosphonium-hexafluoro-phosphate (PyBOP) and NovaSyn TGR resin were purchased from NovaBiochem (Läufelfingen, Switzerland) and GL Biochem Shanghai Ltd (Shanghai, China). Scavengers, coupling agents and cleavage reagents (triisopropylsilane (TIS), 4-methylmorpholine (NMM), piperidine, 1,8-diazabicyclo[5.4.0]undec-7-ene (DBU), trifluoroacetic acid (TFA)) were obtained from Sigma-Aldrich Ltd. (St. Louis, MO, USA). *N*,*N*-dimethylformamide (DMF) and acetonitrile were purchased from Acros Organics (Geel, Belgium), while ethanol and diethyl ether were from Riedel deHäen (Seelze, Germany). All reagents and solvents were of analytical grade or highest available purity.

Peptide Biotin-GGGGGVHHQKLVFFAEDVGSNKGAIIGLMVGGVV-NH_2 (Biotin-G_5-Aβ(12-40)) was synthesized on a NovaSyn TGR resin (0.23 mmol/g coupling capacity) by 9-fluorenylmethoxycarbonyl/tert-butyl (Fmoc/*t*Bu) strategy, using a semiautomated Peptide Synthesizer EPS-221 (ABIMED, Langenfeld, Germany). The following Fmoc-protected amino acid derivatives were employed: Fmoc-Val-OH, Fmoc-Gly-OH, Fmoc-Met-OH, Fmoc-Leu-OH, Fmoc-Ile-OH, Fmoc-Ala-OH, Fmoc-Lys(Boc)-OH, Fmoc-Asn(Trt)-OH, Fmoc-Ser(*t*Bu)-OH, Fmoc-Asp(O*t*Bu)-OH, Fmoc-Glu(O*t*Bu)-OH, Fmoc-Phe-OH, Fmoc-Gln(Trt)-OH and Fmoc-His(Trt)-OH. The protocol of the synthesis was as follows: (i) DMF washing (3 × 1 min); (ii) Fmoc deprotection for 15 min using 2% DBU and 2% piperidine in DMF; (iii) DMF washing (6 × 1 min); (iv) double coupling of 5 equiv of Fmoc-amino acid/PyBOP/NMM in DMF for 45 min; (v) DMF washing (3 × 1 min). After completion of the synthesis, the N-terminal α-amino group was biotinylated using 5 equiv of D-(+)-Biotin/PyBOP/NMM in DMF. The peptide was then cleaved from the resin at 25 °C for 2.5 h using a mixture of TFA, triisopropylsilane and deionized water (95: 2.5: 2.5, v/v/v). The crude product was precipitated with cold diethyl ether, washed three times with diethyl ether and solubilized in 5% aqueous acetic acid prior to freeze-drying. The crude peptide was purified

by reverse phase-high performance liquid chromatography (RP-HPLC) on a semipreparative C_4 column. Purified peptide was characterized by analytical RP-HPLC and matrix assisted laser desorption ionization-Fourier transform ion cyclotron resonance mass spectrometry (MALDI-FTICR MS).
